# Contemporary Clinical Practices in Anticoagulation Management During Cardiopulmonary Bypass: A Europe-Wide Survey

**DOI:** 10.1093/icvts/ivag059

**Published:** 2026-03-05

**Authors:** Rafael Maniés Pereira, Nuno Guerra, Ricardo Ferreira, Ângelo Nobre, Luís Ferreira Moita, Tiago Rodrigues Velho

**Affiliations:** Cardiothoracic Surgery Research Unit, Centro Cardiovascular da Universidade de Lisboa (CCUL@RISE), Faculdade de Medicina da Universidade de Lisboa, Av. Prof Egas Moniz Edifício Reynaldo dos Santos, Lisbon, 1649-028, Portugal; Center for Disease Mechanisms Research, Faculdade de Medicina da Universidade de Lisboa, Av. Prof Egas Moniz Edifício Reynaldo dos Santos, Lisbon, 1649-028, Portugal; Escola Superior de Saúde da Cruz Vermelha Portuguesa, Lisbon, Av. de Ceuta 1, Lisbon, 1300-125, Portugal; Cardiothoracic Surgery Department, Hospital de Santa Maria, Centro Hospitalar Universitário Lisboa Norte, EPE, Av. Prof Egas Moniz, Lisbon, 1649-028, Portugal; Cardiothoracic Surgery Department, Hospital de Santa Maria, Centro Hospitalar Universitário Lisboa Norte, EPE, Av. Prof Egas Moniz, Lisbon, 1649-028, Portugal; Cardiothoracic Surgery Department, Hospital de Santa Maria, Centro Hospitalar Universitário Lisboa Norte, EPE, Av. Prof Egas Moniz, Lisbon, 1649-028, Portugal; Center for Disease Mechanisms Research, Faculdade de Medicina da Universidade de Lisboa, Av. Prof Egas Moniz Edifício Reynaldo dos Santos, Lisbon, 1649-028, Portugal; Cardiothoracic Surgery Research Unit, Centro Cardiovascular da Universidade de Lisboa (CCUL@RISE), Faculdade de Medicina da Universidade de Lisboa, Av. Prof Egas Moniz Edifício Reynaldo dos Santos, Lisbon, 1649-028, Portugal; Center for Disease Mechanisms Research, Faculdade de Medicina da Universidade de Lisboa, Av. Prof Egas Moniz Edifício Reynaldo dos Santos, Lisbon, 1649-028, Portugal; Cardiothoracic Surgery Department, Hospital de Santa Maria, Centro Hospitalar Universitário Lisboa Norte, EPE, Av. Prof Egas Moniz, Lisbon, 1649-028, Portugal

**Keywords:** cardiac surgery, cardiopulmonary bypass, anticoagulation, protamine, activated clotting time

## Abstract

**Objectives:**

Clinical practice in anticoagulation management, particularly in heparin administration, monitoring, reversal and haemostasis, is known to differ significantly. To better characterize this variability, we conducted a Europe-wide survey aimed at mapping current practices, identifying areas of consensus and divergence and guiding future research and standardization efforts.

**Methods:**

A 27-question electronic questionnaire was designed by an expert panel and distributed across European cardiac surgery centres with a snowball sampling method. Results were examined via descriptive statistics, with categorical variables shown as percentages and 95% CIs.

**Results:**

A total of 114 centres from 29 countries completed the questionnaire between February and April 2025. Most centres were high-volume institutions (>500 cases/year, 59.6%) and reported the use of written heparinization protocols (78.1%)—initial heparin dose of 300 IU/kg in 61.4%. Many used normothermic perfusion (52.6%). Pre-cardiopulmonary bypass activated clotting time (ACT) targets varied from 400 s (41.2%) to 480 s (38.6%). There was significant heterogeneity in reversal practices: heparin: protamine ratios were 1:1 in 57.0%, <1:1 in 36.8%, and >1:1 in 6.1%. Universal post-reversal ACT target was absent in 70.2%, with 78.1% using an ACT value close to baseline. Although almost all centres (90.4%) had viscoelastic testing, clinical criteria alone were used in 48.2% to guide transfusion decisions. Only 83.3% of centres had any explicit protocol for managing high-bleeding-risk patients.

**Conclusions:**

While essential heparinization practices demonstrate consensus across Europe, heterogeneity exists in anticoagulation reversal strategies and haemostasis monitoring. However, significant variability remains in protamine dosing, post-reversal monitoring, and the use of viscoelastic assays, representing an opportunity to optimize patient care.

## Graphical abstract

**Figure:**
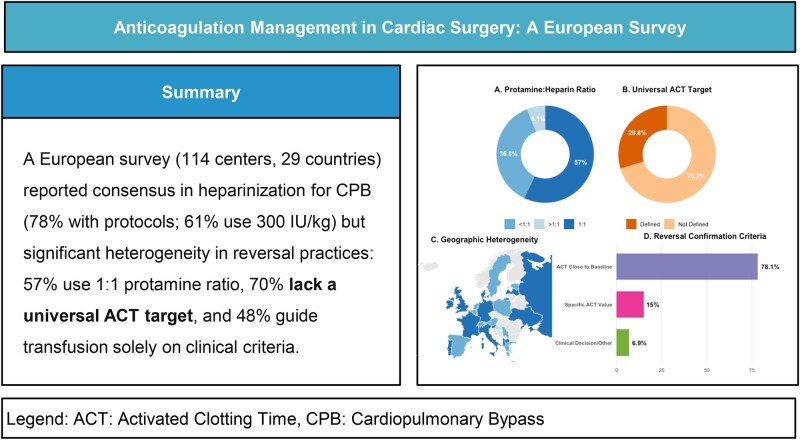

## INTRODUCTION

Systemic heparin anticoagulation is a cornerstone of safe adult cardiac surgery with cardiopulmonary bypass (CPB), playing an essential role in preventing potentially devastating thrombosis within the extracorporeal circuit. Monitoring of anticoagulation during CPB has traditionally been controlled by the Activated Clotting Time (ACT), a point-of-care functional assay that remains the gold standard in most operating rooms worldwide. The central challenge lies in achieving a delicate balance: providing sufficient anticoagulation to safely maintain CPB without increasing the risk of bleeding complications.

Recommendations from international guidelines, including those from the European Association for Cardio-Thoracic Surgery (EACTS) and the European Association of Cardiothoracic Anaesthesiology and Intensive Care (EACTA), provide a framework for best practices, such as a recommended minimum ACT of 480 seconds to adequately inhibit thrombin generation during CPB.^1^^,^^2^ However, despite these recommendations, widespread recognition of significant variability in clinical practice. This variability extends beyond initial heparin dosing and ACT targets, including more complex and critical aspects of care, such as management of heparin resistance, strategies for protamine reversal, assessment of adequate haemostasis after reversal, and integration of the use of advanced viscoelastic monitoring techniques like thromboelastography or rotational thromboelastometry (TEG/ROTEM).

Such variation likely reflects a combination of historical precedent, institutional practice, and a relative lack of high-quality evidence to guide many specific therapeutic decisions. While the underlying principles of anticoagulation management are generally shared, the absence of standardized protocols, particularly in the post-reversal phase, may potentially impact patient outcomes and affect resource utilization. To date, no comprehensive assessment has described current European practices in this field.

Therefore, to address this gap, we conducted a Europe-wide survey aimed at defining contemporary clinical practices in anticoagulation management during CPB. The primary outcome was to map the spectrum of practices related to heparin administration, ACT monitoring, protamine reversal, and haemostasis management. Our goal was to identify areas of broad consensus and significant heterogeneity, thereby informing future research priorities and efforts towards standardization.

## METHODS

### Survey design

This study was approved by the Ethics Committee of Centro Académico de Medicina de Lisboa (Reference No 386/21 on March 17, 2022). An expert panel composed of perfusionists, and cardiac surgeons was assembled to develop the survey. The questionnaire was created through a structured process of item generation and reduction, followed by successive rounds of refinement, incorporating feedback from both local and European experts who were independent of the development team, to ensure clarity, and content and face validity.

The questionnaire was designed to capture information across the following domains: (1) heparin/ACT monitoring; (2) heparin doses and protocols; (3) protamine and anticoagulation reversal; (4) post-reversal monitoring; (5) additional protamine doses; (6) application of advanced technologies (TEG/ROTEM); (7) clinical decision-making and protocols. The questionnaire consisted predominantly of closed items with predefined categorical response options. A limited number of open optional fields have been included to allow respondents to specify additional details or clarify local practices where applicable. Likert-type scales were not used.

Centre-specific characteristics included the scope of practice and the annual volume of procedures with CPB, self-reported by the respondents. Where applicable, open-field responses were normalized into pre-defined clinical categories (eg, groupings of ACT devices, measurement intervals and ACT targets; classification of protamine strategies), ensuring meaningful comparison across centres. For conditional items (eg, “Yes” responses requiring further detail), follow-up questions were included to clarify clinical practices and underlying rationale.

The final version of the questionnaire was approved by the study’s steering committee and disseminated prospectively through professional networks in perfusion and cardiac surgery across Europe, using a convenience and snowball sampling strategy. Distribution included direct invitations by email, circulation through professional mailing lists, and sharing between clinicians and perfusionists. Participation was open to centres in all European countries, with responses received on a voluntary basis.

Responses were obtained between February and April 2025. The full questionnaire is presented in Supplementary Material 1. The reporting of this internet-based survey followed the principles of the Checklist for Reporting Results of Internet E-Surveys guidelines where applicable.

### Data analysis

Categorical variables were described as absolute numbers and percentages, calculated using the number of valid responses for each item as the denominator. Proportions are presented with 95% CI, computed using the Agresti–Coull method. Comparisons between categories were performed with the chi-squared test; when expected counts were inferior to 5 in any cell, Fisher’s exact test was used.

There were no primary continuous variables; free numerical responses (eg, target ACT values) were harmonized into clinically used categories (400, 420, 440, 450, 480 seconds), as per the questionnaire. During data cleansing, responses were analysed for internal logical consistency, particularly for open-ended response items. These responses (eg, ACT device, strategies for high bleeding risk) were normalized by pre-specified rules [grouping, for example, Hemochron (Werfen), Medtronic ACT/ACT+, HMS/Hepcon (Medtronic), Helena/Actalyke, i-STAT (Abbott), and Other/Not specified. ACT measurement frequency during CPB was recoded in groups, considering: “every 30 min,” “every 60 min,” “<30 min,” “based on clinical need,” and “other.”

High-volume centres were defined a priori as those performing >500 surgeries with CPB per year, according to the questionnaire’s category. For frequency-based questions, responses were kept in the pre-defined categorical ranges of the instrument.

“Not reported” values were included in the description but treated as missing data in inferential analyses, applying model-wise complete-case analysis. Categories with a very small sample were, when clinically justifiable, collapsed to ensure the stability of the estimates. All analyses were 2-tailed, with a significance level of α = 0.05. The Benjamini-Hochberg procedure (FDR) was applied to control for multiplicity in secondary analyses. The analysis was performed in R (RStudio 4.5.1)^3^ with the *ggplot2*,^4^  *rnaturalearth*,^5^  *rnaturalearthdata*,^6^ and dplyr^7^ packages.

## RESULTS

**Figure 1.**
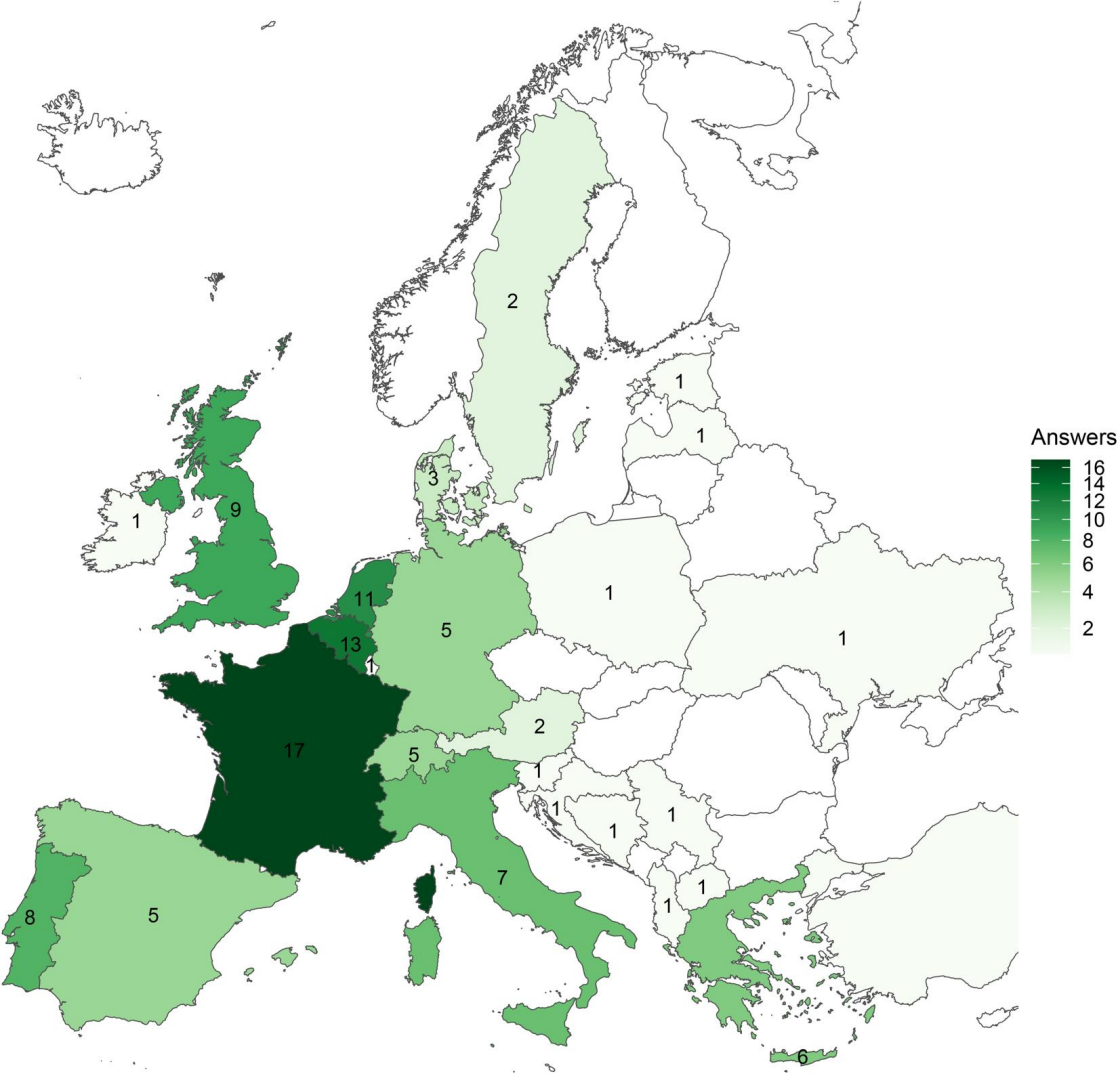
Choropleth Map Representing the Geographic Distribution and Total Number of Responses Received from Each Country Represented in the Survey

A total of 114 responses were received from 29 countries. Geographic distribution of responses is illustrated in the choropleth map (Figure 1), highlighting the widespread participation across Europe. A higher concentration of responses was observed in Western Europe, with additional contributions from multiple countries in Central, Nordic, and Eastern Europe. Most centres reported performing more than 500 surgeries per year (59.6%). Normothermia (>35 °C) was the most frequently reported temperature management strategy during CPB (52.6%). A written protocol for heparinization was reported by 89 centres (78.1%), while 25 (21.9%) indicated that no such protocol existed. The findings are described in Table 1.

**Table 1.** Characteristics of the Responding Centres

Question/response	*N*	Percent (%)	95% CI
**Number of on-pump heart surgeries performed annually in your hospital?**			
>500	68	59.6	(50.5-68.2)
301-500	28	24.6	(17.5-33.2)
100-300	17	14.9	(9.4-22.7)
<100	1	0.9	(0.0-5.3)
**Is the surgery performed with normothermic or hypothermic circulation?**			
Normothermia (>35 °C)	60	52.6	(43.5-61.6)
Mild hypothermia (32-35 °C)	51	44.7	(35.9-53.9)
Moderate hypothermia (28-32 °C)	3	2.6	(0.6-7.8)
**Is there a written protocol in your hospital centre where heparinization is described?**			
Yes	89	78.1	(69.6-84.7)
No	25	21.9	(15.3-30.4)

### Heparin protocols and initial dose

**Figure 2.**
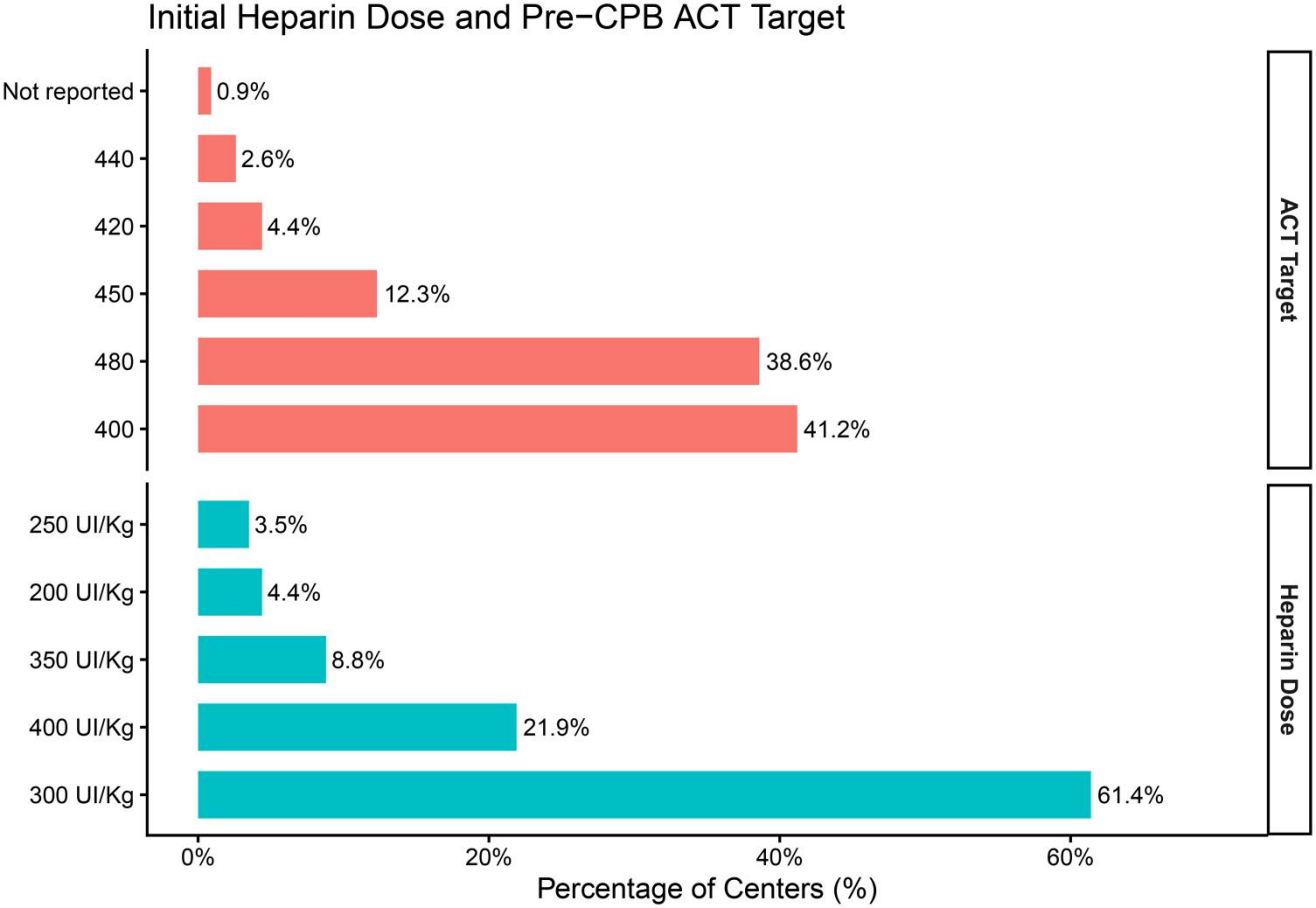
Percentage (%) of Centres by Category of Preoperative Cardiopulmonary Bypass (CPB) Activated Clotting Time (ACT) Target and Initial Heparin Dose

The most used initial heparin dose prior to CPB was 300 IU/kg (61.4%; *n* = 70), followed by 400 IU/kg (21.9%; *n* = 25) and 350 IU/kg (8.8%; *n* = 10). Pre-CPB target ACT values varied, with the most frequently cited targets being 400 seconds (41.2%; *n* = 47) and 480 seconds (38.6%; *n* = 44). These data are illustrated in Figure 2.

### ACT and heparin level monitoring

During CPB, ACT monitoring practices varied among centres. The majority (72.8%, *n* = 83) reported measuring ACT every 30 minutes, while 11.4% (*n* = 13) did so every 60 minutes, and 8.8% (*n* = 10) at intervals shorter than 30 minutes. ACT remained the predominant method for monitoring heparin levels, used by 93.9% of the centres (*n* = 107). A minority (5.3%, *n* = 6) used heparin concentration-based monitoring systems, such as Hepcon HMS. Regarding the devices used for ACT measurement, the most reported was the Hemochron (Werfen) system (54.4%; *n* = 62), followed by the Medtronic ACT/ACT+ (14.0%; *n* = 16) and the Medtronic HMS (7.0%; *n* = 8).

### Strategies in high-bleeding-risk patients and heparin resistance

Most centres reported having no specific protocol for managing high-risk bleeding patients (83.3%; *n* = 95). Among the centres that did report targeted approaches, the most commonly cited strategies included the use of TEG/ROTEM for coagulation monitoring (5.3%; *n* = 6) and administration of antifibrinolytics (2.6%; *n* = 3). In the event of suspected heparin resistance, the most frequently reported initial intervention was the use of antithrombin III (AT-III) (52.6%; *n* = 60), followed by increasing the heparin dose (27.2%; *n* = 31).

### Protamine: Calculation, administration, and safety

Protamine dosing adjustment was most frequently based on the total amount of heparin administered (68.4%; *n* = 78). The reported heparin: protamine dosing ratio was 1:1 in 57.0% of centres (*n* = 65), less than 1:1 in 36.8% (*n* = 42), and greater than 1:1 in 6.1% (*n* = 7). Protamine was primarily administered either as a slow infusion (41.2%; *n* = 47) or as a slow diluted infusion (37.7%; *n* = 43). The frequency of protamine-related complications was reported as very low in most centres (85.1%; *n* = 97) and low in an additional 13.2% (*n* = 15). Post-protamine ACT measurement was typically performed after a waiting period of 5 minutes (47.4%, *n* = 54), while 38.6% (*n* = 44) reported waiting longer than 5 minutes.

### Post-reversal monitoring and targets

**Figure 3.**
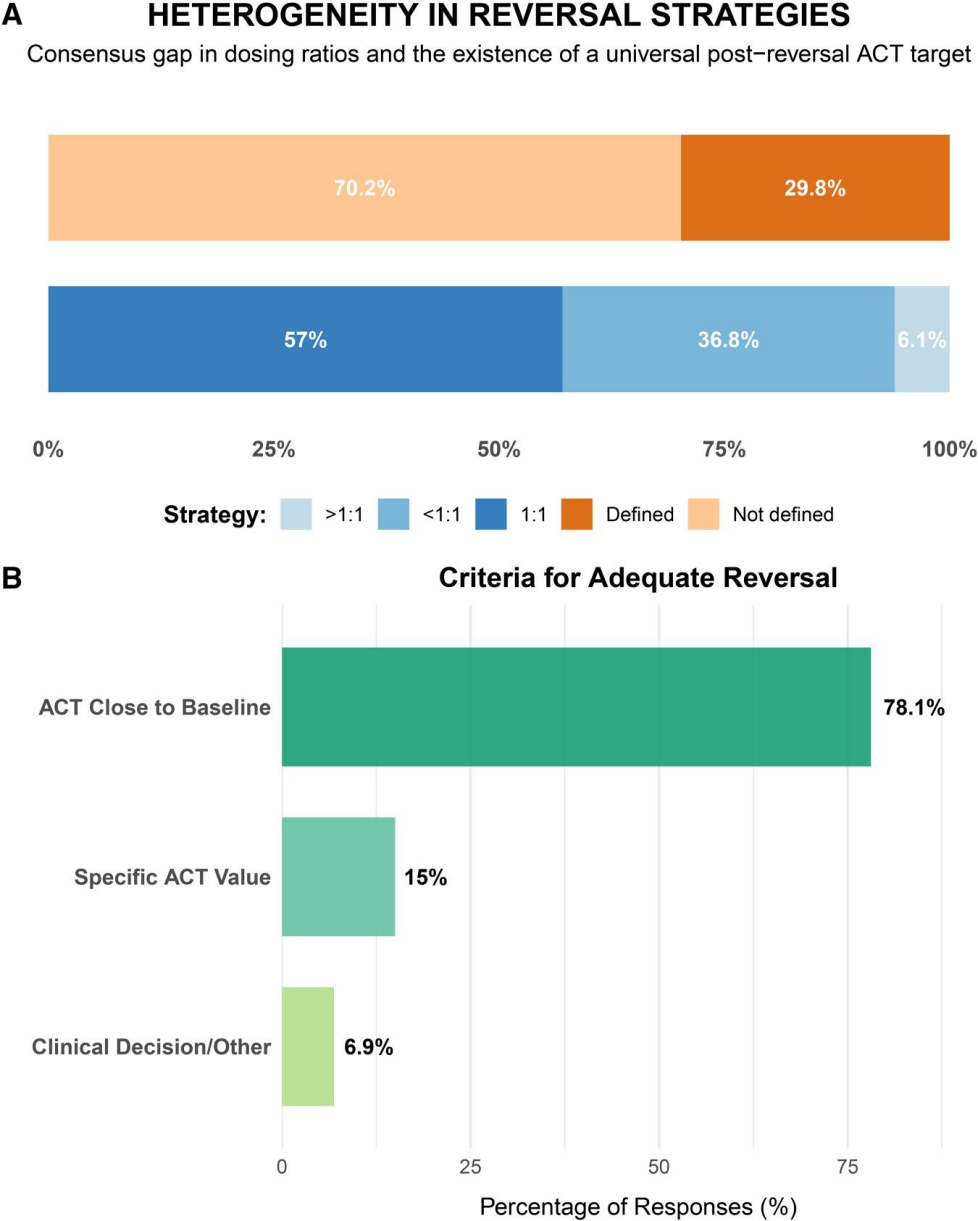
(A) Percentage of Centres with a Defined Vs Undefined Heparin Reversal Protocol, Including the Distribution of Heparin-to-Protamine Dosing Ratios Used; (B) Percentage of Responses Indicating the Criteria Used to Define Adequate Heparin Reversal Abbreviation: ACT, activated clotting time.

**Figure 4.**
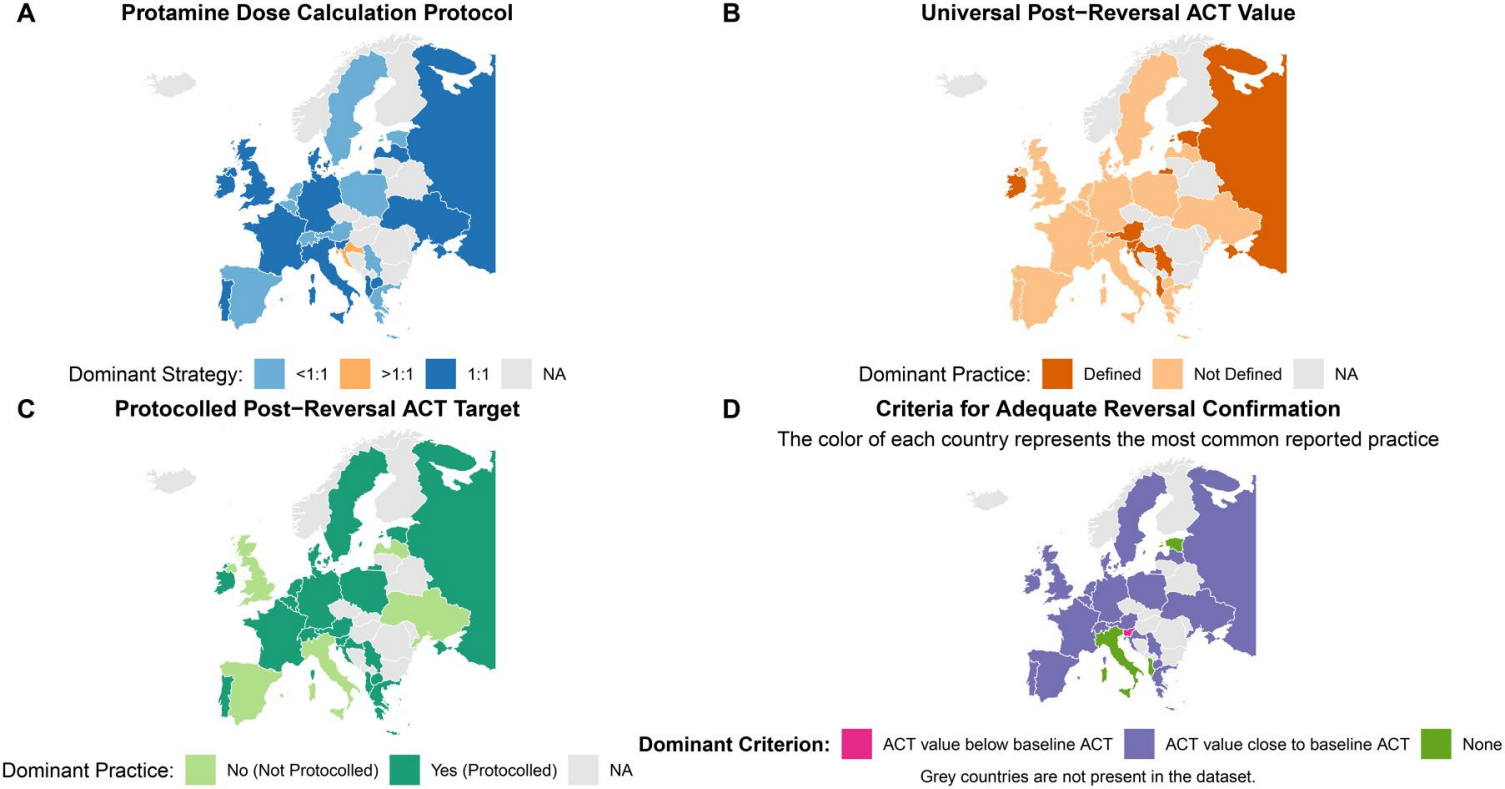
Geographical Variability of Protamine Reversal Practices Across European Cardiac Surgery Centres (A) Protamine dose calculation protocol (heparin:protamine ratio). (B) Existence of a universal post-reversal ACT value. (C) Existence of a protocolled universal post-reversal ACT value. (D) Dominant criterion for adequate assessment of heparin reversal. The colour of each country represents the most common practice reported by centres from that nationality. grey countries are not represented in the dataset. Abbreviations: ACT, activated clotting time; NA, not available.

A protocolized post-reversal ACT target was reported by 71.9% of centres (*n* = 82). Among these, the majority (70.2%; *n* = 80) did not define a single universal value for all patients. The most used criterion to assess adequate heparin reversal was achieving an ACT “close to baseline,” as reported by 78.1% of centres (*n* = 89) (these data are summarized in Figure 3). The heterogeneity in protamine reversal practices was further analysed from a geographical perspective (Figure 4). Significant variability was observed across European countries regarding both the protamine dose calculation protocol (Figure 4A) and the definition of a universal post-reversal ACT value (Figure 4B and C). The “*ACT close to baseline*” criteria emerged as the dominant practice in most countries to assess adequate heprin reversal (Figure 4D).

### Additional protamine doses

A total of 82.5% of centres (*n* = 94) reported they would consider administering an additional dose of protamine under specific values/conditions. The main reason was difficulty achieving haemostasis (58.8%; *n* = 67), followed by an isolated ACT value (26.3%; *n* = 30). Regarding dosing strategies for the booster dose, 21.9% of the centres (*n* = 25) reported using a specific fixed additional dose, 7.0% (*n* = 8) adjusted the dose by ACT values, and 4.4% (*n* = 5) left the decision to clinician discretion. Notably, 47.4% of respondents did not provide information on this specific question.

### Advanced technologies, transfusion decisions, and recent protocol changes

Most centres reported having access to viscoelastic testing with TEG/ROTEM (90.4%; *n* = 103). Among these, the device was in the operating room in 50.0% of cases (*n* = 57), while 40.4% (*n* = 46) reported availability in another department. The decision to administer blood products varied and was based solely on clinical criteria in 48.2% of centres (*n* = 55), exclusively on TEG/ROTEM results in 37.7% (*n* = 43), and using a combined clinical and viscoelastic-guided approach in 8.8% (*n* = 10). Most centres (86.8%; *n* = 99) reported no recent implemented changes to their ACT/protamine protocols based on new evidence. Among the minority that had implemented changes (13.2%; *n* = 15), the most frequently mentioned modification was an adjustment in protamine dose (4.4%; *n* = 5).

The full category breakdown is provided in **Table S1**.

## DISCUSSION

This European survey provides a comprehensive overview of contemporary practices in anticoagulation management during cardiac surgery with CPB in adults. With 114 responses from 29 European countries, our study reveals a landscape of both established consensus and significant variability in the clinical management of perioperative anticoagulation, reflecting the complexity and lack of universal standards in the field. Our primary finding is that, while a core set of practices, such as the use of written heparinization protocols, an initial heparin dose of 300 IU/kg, and pre-CPB ACT targets of 400-480 seconds, are well-established, substantial variability persists in the critical phases of anticoagulation reversal and haemostasis management. This suggests that while foundational safety standards are widely adopted, optimizing care at the end of the procedure with CPB, particularly in complex scenarios, remains an area of ongoing debate and institutional preference.

Interestingly, we observed a high prevalence of written heparinization protocols (78.1%) and the common use of a 300 IU/kg heparin bolus, demonstrating a strong common ground in the fundamental principles of sabe CPB conduct in clinical practice. Similarly, the reported pre-CPB ACT targets represent a widespread practical consensus, predominantly ranging within 400-480 seconds. The observed strategy aligns with the general recommendations from major clinical European associations, such as EACTS and EACTA, which consider an ACT over 480 seconds a safe threshold, particularly when using uncoated circuits.^1^ However, as these same guidelines acknowledge, there is no single universally accepted target value due to the known variability in CPB circuits and ACT measurement assays. However, information regarding circuit surface coating was not systematically collected in this survey, precluding differentiation of ACT targets according to circuit type. Therefore, the observed consistency in our survey is more related to a pragmatic convergence shaped by clinical experience, rather than the successful implementation of a single, clear directive. The persistence of higher ACT thresholds across centres may reflect a combination of safety considerations, institutional protocols, historical practice patterns, and generational inheritance, rather than exclusive adherence to the most recent evidence or to manufacturer-specific instructions for use of coated circuits. This finding underscores the complexity of anticoagulation management during CPB and highlights the need for future studies that integrate circuit characteristics when evaluating anticoagulation strategies.

In contrast, our data reveal significant heterogeneity in strategies for heparin reversal with protamine. While most centres (68.4%) calculate the protamine dose based on the total heparin administered, there is a lack of a standardized heparin: protamine ratio. Thus, 57% of the centres use a 1:1 ratio, while 37% use a ratio of less than 1:1, highlighting a critical area of clinical uncertainty. This variability is clinically relevant, as both underdosing and overdosing of protamine are associated with adverse outcomes, including rebound anticoagulation and coagulopathy, respectively.^8^^,^^9^ This variability persists despite contemporary European patient blood management guidelines recommending protamine reversal strategies using ratios lower than 1:1, supported by a Class I recommendation with Level of Evidence B. Therefore, the observed heterogeneity should not be interpreted as reflecting uncertainty in the underlying scientific evidence. Instead, it highlights a gap between evidence-based recommendations and real-world clinical practice, with a substantial proportion of centres continuing to apply higher or fixed reversal ratios. This discrepancy highlights challenges in implementing guidelines on anticoagulation management during CPB.

Furthermore, the ACT value after protamine reversal has a good predictive value for the use of transfusion and postoperative bleeding, with values above 140 seconds being associated with higher risk of transfusion and increased bleeding.^10^ Thus, more evidence is needed to recommend and support an ideal ACT value after protamine administration, especially since most centres (70.2%) lack a defined post-reversal ACT target, creating a clinical grey zone where adequacy of reversal is judged subjectively, often guided by inaccurate visual assessment of haemostasis rather than objective metrics. This gap underscores the need for more research in the field, potentially advocating for individualized approaches based on heparin concentration measurements or the integration of recent technologies like ROTEM, which have been shown to be superior to fixed-dose ratios in reducing bleeding and transfusion needs.^11–14^ As an example, ROTEM has been shown to be effective in detecting residual heparin after protamine administration and is more reliable than ACT as a predictor of heparin rebound.^15^

Another area of variability is the management of heparin resistance and patients at high risk of bleeding. While the administration of antithrombin-III is the most common strategy for heparin resistance (52.6%), a significant proportion of centres (27.2%) still opt to increase the heparin dose, a strategy that can be ineffective in cases of true antithrombin deficiency. However, the most concerning finding is that most centres (83.3%) do not have a specific protocol for managing high-bleeding risk patients. This highlights an area for further development in proactive haemostasis management and the implementation of patient blood management programs, which are strongly recommended by leading clinical associations.^2^^,^^13^^,^^14^^,^^16^

Our survey also uncovered inconsistencies in the adoption of advanced technologies, as briefly discussed earlier in this section. Despite the wide availability of viscoelastic testing (eg, TEG/ROTEM) in over 90% of centres, integration into clinical decision-making remains inconsistent. In nearly half of the centres (48.2%), the decision to use blood products was based exclusively on clinical criteria, highlighting the encountered gap between the available technology and its integration into goal-directed transfusion algorithms. The available data robustly supports the use of viscoelastic testing to guide the use of blood products, consistently showing reductions in transfusions and re-exploration rates, which significantly impacts peri-operative morbidity and mortality, and healthcare-associated costs.^2^^,^^17^^,^^18^ Barriers to broader adoption may include cost, training difficulties, and a lack of institutional protocols, representing a key field for quality improvement in Europe.

Rather than establishing normative consensus, the findings of this survey should be interpreted as a descriptive mapping of current clinical practice. Areas where real-world practice diverges from contemporary guideline recommendations, such as ACT targets or protamine reversal ratios, highlight gaps in implementation rather than uncertainty in the underlying evidence. These observations should be considered hypothesis-generating and provide a framework for future research aimed at understanding the drivers of deviation from guidelines, including circuit characteristics, institutional experience, and risk perception.

Finally, the observation that most of centres (86.8%) have not recently updated their anticoagulation protocols should not be interpreted necessarily as an indicator of clinical inertia. Instead, it may reflect a broader challenge issue: the scarcity of high-impact, practice-changing research in the field. Without robust evidence from large-scale randomized trials, many decisions, particularly those related to protamine reversal and subsequent haemostasis management, continue to rely essentially on institutional experience and expert opinion. This evidence gap contributes to the heterogeneity identified in our survey and highlights the critical need for further research to build the foundation required for standardized, evidence-based and optimized patient care.

### Limitations

This study has several limitations inherent to its survey-based design. The use of a convenience and snowball sampling technique may have introduce selection bias, potentially over-representing centres with higher academic engagement. Furthermore, the responses reflect self-reported practices, which may not always entirely correlate with real-world clinical conduct.

An additional methodological limitation relates to the development of the questionnaire. A formal consensus-building approach, such as a Delphi methodology, was not employed. However, the primary objective of this survey was descriptive, aiming to capture real-world contemporary practices rather than to generate consensus recommendations. To balance methodological rigor with feasibility and broad European participation, the questionnaire was developed by a multidisciplinary expert panel and refined through successive rounds of feedback, including input from independent European experts, to ensure clarity, relevance, and face validity of the items.

The response rate could not be calculated, as the total number of institutions reached through open dissemination is unknown. No formal a priori sample size calculation or predefined target number of responses was established, as the survey was designed as an exploratory and descriptive study. Consequently, non-response bias cannot be excluded.

Several additional sources of bias may further limit generalizability. Coverage bias is likely, as dissemination through professional networks may have favoured participation from high-volume or academically engaged centres. Language bias cannot be excluded, as the questionnaire was administered exclusively in English, potentially discouraging participation from some regions. Social desirability bias is also a concern, as respondents may have overreported adherence to written protocols or guideline-consistent practices, particularly regarding anticoagulation management.

Finally, although responses were obtained from 29 European countries, participation was disproportionately higher in Western Europe. Taken together, these limitations indicate that the findings should be interpreted as descriptive of practices among participating centres rather than as representative of all European cardiac surgery institutions.

## CONCLUSIONS

In conclusion, this study highlights a well-stablished core of anticoagulation practices in cardiac surgery with CPB across Europe, particularly regarding heparinization protocols, ACT monitoring, and standardized initial heparin dosing. These core elements reflect a strong consensus and a reassuring level of safety.

However, significant variability remains in post-reversal management, specifically in defining adequate reversal, timing of ACT reassessment, criteria for additional protamine dosing, and the integration of viscoelastic testing into transfusion strategies. These inconsistencies suggest a continued reliance on local experience and underscore a need for greater standardization.

While greater harmonization of anticoagulation reversal practices may represent an opportunity to reduce unwarranted inter-centre variability, such efforts must be guided by outcome-based evidence. Detailed inter-centre comparisons and prospective studies evaluating operative and postoperative outcomes are required to ensure that patient safety is maintained and improved before widespread implementation of standardized protocols.

## Supplementary Material

ivag059_Supplementary_Data

## Data Availability

Research data will be made available upon reasonable request to the corresponding author.
