# Supplementary material for: Contemporary Clinical Practices in Anticoagulation Management During Cardiopulmonary Bypass: A Europe-Wide Survey
Source: Interdiscip Cardiovasc Thorac Surg. 2026 Mar 5;41(3):ivag059. doi: 10.1093/icvts/ivag059 (PMC12996909; doi:10.1093/icvts/ivag059)
Supplement: ivag059_Supplementary_Data [file ivag059_supplementary_data.zip › Supplementary Table S1.docx]

**Supplementary Table S1** – Item-wise survey responses: absolute counts (N), relative frequencies (%), and 95% confidence intervals (Agresti–Coull method).

| **Question/Response** | **N** | **Percent** | **CI 95%** |
| --- | --- | --- | --- |
| **What is the device used for monitoring ACT?** | | | |
| Hemochron (Werfen) | 62 | 54.4% | (45.2% - 63.2%) |
| Other/Not specified | 19 | 16.7% | (10.9% - 24.7%) |
| Medtronic ACT/ACT+ | 16 | 14.0% | (8.7% - 21.7%) |
| HMS (Medtronic) | 8 | 7.0% | (3.4% - 13.4%) |
| Helena/Actalyke | 6 | 5.3% | (2.2% - 11.2%) |
| i-STAT (Abbott) | 3 | 2.6% | (0.6% - 7.8%) |
| **Do you use any device for quantifying the dose-response of heparin?** | | | |
| No | 86 | 75.4% | (66.8% - 82.5%) |
| Yes | 28 | 24.6% | (17.5% - 33.2%) |
| **What dose of heparin is routinely administered before starting CPB?** | | | |
| 300 UI/Kg | 70 | 61.4% | (52.2% - 69.8%) |
| 400 UI/Kg | 25 | 21.9% | (15.3% - 30.4%) |
| 350 UI/Kg | 10 | 8.8% | (4.7% - 15.6%) |
| 200 UI/Kg | 5 | 4.4% | (1.6% - 10.1%) |
| 250 UI/Kg | 4 | 3.5% | (1.1% - 9.0%) |
| **What is the target value of ACT after heparin administration and before on-pump entry?** | | | |
| 400 seconds | 47 | 41.2% | (32.6% - 50.4%) |
| 480 seconds | 44 | 38.6% | (30.2% - 47.8%) |
| 450 seconds | 14 | 12.3% | (7.3% - 19.7%) |
| 420 seconds | 5 | 4.4% | (1.6% - 10.1%) |
| 440 seconds | 3 | 2.6% | (0.6% - 7.8%) |
| Not reported | 1 | 0.9% | (0.0% - 5.3%) |
| **How often do you measure ACT during CPB?** | | | |
| Every 30 minutes | 83 | 72.8% | (64.0% - 80.2%) |
| Every 60 minutes | 13 | 11.4% | (6.7% - 18.7%) |
| Less than 30 minutes | 10 | 8.8% | (4.7% - 15.6%) |
| When clinically indicated | 5 | 4.4% | (1.6% - 10.1%) |
| Other fixed interval | 2 | 1.8% | (0.1% - 6.6%) |
| Variable interval | 1 | 0.9% | (0.0% - 5.3%) |
| **What method do you use to monitor heparin levels during CPB?** | | | |
| Activated Clotting Time (ACT) | 107 | 93.9% | (87.7% - 97.2%) |
| Heparin concentration (e.g., Hepcon HMS) | 6 | 5.3% | (2.2% - 11.2%) |
| Others | 1 | 0.9% | (0.0% - 5.3%) |
| **Does the team follow any specific strategy for managing heparin in high-risk bleeding patients (e.g., renal failure, redo surgery, minimally invasive cardiac surgery)?** | | | |
| No | 95 | 83.3% | (75.3% - 89.1%) |
| Yes | 19 | 16.7% | (10.9% - 24.7%) |
| **If you answered yes to the previous question, please specify?** | | | |
| Not answered | 95 | 83.3% | (75.3% - 89.1%) |
| TEG/ROTEM monitoring | 6 | 5.3% | (2.2% - 11.2%) |
| Antifibrinolytics | 3 | 2.6% | (0.6% - 7.8%) |
| Adjuvant therapies | 2 | 1.8% | (0.1% - 6.6%) |
| HMS protocols | 2 | 1.8% | (0.1% - 6.6%) |
| Individualized management | 2 | 1.8% | (0.1% - 6.6%) |
| No specific strategy | 2 | 1.8% | (0.1% - 6.6%) |
| Surgical strategies | 2 | 1.8% | (0.1% - 6.6%) |
| **What is the strategy for suspected heparin resistance (e.g., reduced antithrombin III levels)?** | | | |
| Administration of AT-III | 60 | 52.6% | (43.5% - 61.6%) |
| Increased heparin dose | 31 | 27.2% | (19.8% - 36.0%) |
| No specific protocol | 17 | 14.9% | (9.4% - 22.7%) |
| Use of bivalirudin or another alternative anticoagulant | 6 | 5.3% | (2.2% - 11.2%) |
| **Is the protamine dose adjusted according to any specific variable?** |  |  |  |
| Total heparin dose administered | 78 | 68.4% | (59.4% - 76.3%) |
| Fixed dose based on protocol | 17 | 14.9% | (9.4% - 22.7%) |
| Patient weight | 15 | 13.2% | (8.0% - 20.7%) |
| CPB duration | 4 | 3.5% | (1.1% - 9.0%) |
| **What is the protocol for calculating the dose of protamine administered after weaning from CPB?** | | | |
| 01:01 | 65 | 57.0% | (47.8% - 65.7%) |
| <1:1 | 42 | 36.8% | (28.5% - 46.0%) |
| >1:1 | 7 | 6.1% | (2.8% - 12.3%) |
| **How is protamine administered?** | | | |
| Slow infusion | 47 | 41.2% | (32.6% - 50.4%) |
| Slow infusion (diluted) | 43 | 37.7% | (29.4% - 46.9%) |
| Bolus | 14 | 12.3% | (7.3% - 19.7%) |
| Case-dependent | 10 | 8.8% | (4.7% - 15.6%) |
| **What is the frequency of complications related to protamine administration at your center (e.g., severe hypotension, anaphylactic reaction, pulmonary hypertension)?** | | | |
| Very low | 97 | 85.1% | (77.3% - 90.6%) |
| Low | 15 | 13.2% | (8.0% - 20.7%) |
| Moderate | 2 | 1.8% | (0.1% - 6.6%) |
| **How long do you wait after administering protamine before collecting the blood sample for ACT measurement?** | | | |
| 5 minutes | 54 | 47.4% | (38.4% - 56.5%) |
| > 5 minutes | 44 | 38.6% | (30.2% - 47.8%) |
| < 5 minutes | 16 | 14.0% | (8.7% - 21.7%) |
| **Is the target ACT value protocolled after reversal with protamine?** | | | |
| Yes | 82 | 71.9% | (63.0% - 79.4%) |
| No | 32 | 28.1% | (20.6% - 37.0%) |
| **Is there any universal numerical value of ACT to be achieved by all patients after reversal with protamine (e.g., ACT < 140 seconds)?** | | | |
| No | 80 | 70.2% | (61.2% - 77.8%) |
| Yes | 34 | 29.8% | (22.2% - 38.8%) |
| **If you answered yes to the previous question, what is this value?** | | | |
| Not answered | 81 | 71.1% | (62.1% - 78.6%) |
| Specific absolute value | 16 | 14.0% | (8.7% - 21.7%) |
| Based on baseline ACT | 8 | 7.0% | (3.4% - 13.4%) |
| Other approach | 5 | 4.4% | (1.6% - 10.1%) |
| Clinically adjusted value | 1 | 0.9% | (0.0% - 5.3%) |
| HMS protocol based | 1 | 0.9% | (0.0% - 5.3%) |
| No universal value | 1 | 0.9% | (0.0% - 5.3%) |
| TEG/ROTEM guided | 1 | 0.9% | (0.0% - 5.3%) |
| **Are any of the following options used in your hospital to affirm that the ACT value is complete or satisfactory after reversal with protamine?** | | | |
| ACT value close to baseline ACT | 89 | 78.1% | (69.6% - 84.7%) |
| None | 17 | 14.9% | (9.4% - 22.7%) |
| ACT value below baseline ACT | 8 | 7.0% | (3.4% - 13.4%) |
| **Would you consider administering another dose of protamine at any ACT value?** | | | |
| Yes | 94 | 82.5% | (74.4% - 88.4%) |
| No, an additional dose of protamine is never administered | 20 | 17.5% | (11.6% - 25.6%) |
| **If you answered yes to the previous question, what is this value?** | | | |
| Not answered | 30 | 26.3% | (19.1% - 35.1%) |
| Other criterion | 24 | 21.1% | (14.5% - 29.5%) |
| Based on baseline ACT | 23 | 20.2% | (13.8% - 28.5%) |
| Specific absolute value | 15 | 13.2% | (8.0% - 20.7%) |
| Clinical bleeding assessment | 9 | 7.9% | (4.0% - 14.5%) |
| Device-guided decision | 5 | 4.4% | (1.6% - 10.1%) |
| Specific protamine dose | 5 | 4.4% | (1.6% - 10.1%) |
| Individualized approach | 2 | 1.8% | (0.1% - 6.6%) |
| No specific criterion | 1 | 0.9% | (0.0% - 5.3%) |
| **If you administer an additional dose of protamine, what is the main reason for this decision?** | | | |
| Based on hemostasis difficulties only | 67 | 58.8% | (49.6% - 67.4%) |
| Based on ACT value only | 30 | 26.3% | (19.1% - 35.1%) |
| No specific reason/Not applicable | 14 | 12.3% | (7.3% - 19.7%) |
| Not answered | 3 | 2.6% | (0.6% - 7.8%) |
| **When administering a protamine booster dose, what is the correct dosage to be administered?** | | | |
| Not answered | 54 | 47.4% | (38.4% - 56.5%) |
| Specific fixed dose | 25 | 21.9% | (15.3% - 30.4%) |
| Other approach | 15 | 13.2% | (8.0% - 20.7%) |
| ACT-based variable dosing | 8 | 7.0% | (3.4% - 13.4%) |
| Clinician decision-based | 5 | 4.4% | (1.6% - 10.1%) |
| Not specified/unknown | 4 | 3.5% | (1.1% - 9.0%) |
| Protocol-based dosing | 2 | 1.8% | (0.1% - 6.6%) |
| Other specific approach | 1 | 0.9% | (0.0% - 5.3%) |
| **Does your hospital have a thromboelastogram (TEG or ROTEM)?** | | | |
| Yes | 103 | 90.4% | (83.4% - 94.7%) |
| No | 11 | 9.6% | (5.3% - 16.6%) |
| **If yes, is this device in the operating room or another service (e.g., Blood Service or Analysis Laboratory)?** | | | |
| Operating room | 57 | 50.0% | (41.0% - 59.0%) |
| Another Service | 46 | 40.4% | (31.8% - 49.5%) |
| Not reported | 11 | 9.6% | (5.3% - 16.6%) |
| **How is the decision made to administer blood products?** | | | |
| Clinical criteria only | 55 | 48.2% | (39.3% - 57.3%) |
| TEG/ROTEM only | 43 | 37.7% | (29.4% - 46.9%) |
| Combined methods | 10 | 8.8% | (4.7% - 15.6%) |
| Other approaches | 4 | 3.5% | (1.1% - 9.0%) |
| ACT only | 2 | 1.8% | (0.1% - 6.6%) |
| **Has your hospital recently implemented any changes to ACT/protamine protocols based on new scientific data?** | | | |
| No | 99 | 86.8% | (79.3% - 92.0%) |
| Yes | 15 | 13.2% | (8.0% - 20.7%) |
| **If you answered yes to the previous question, please specify?** | | | |
| Not answered | 98 | 86.0% | (78.3% - 91.3%) |
| Protamine dosage adjustment | 5 | 4.4% | (1.6% - 10.1%) |
| Dose individualization | 2 | 1.8% | (0.1% - 6.6%) |
| Heparin dosage adjustment | 2 | 1.8% | (0.1% - 6.6%) |
| No changes/not applicable | 2 | 1.8% | (0.1% - 6.6%) |
| Other change | 2 | 1.8% | (0.1% - 6.6%) |
| Technology implementation | 2 | 1.8% | (0.1% - 6.6%) |
| Evidence-based change | 1 | 0.9% | (0.0% - 5.3%) |
