# Supplementary material for: Contemporary Clinical Practices in Anticoagulation Management During Cardiopulmonary Bypass: A Europe-Wide Survey
Source: Interdiscip Cardiovasc Thorac Surg. 2026 Mar 5;41(3):ivag059. doi: 10.1093/icvts/ivag059 (PMC12996909; doi:10.1093/icvts/ivag059)
Supplement: ivag059_Supplementary_Data [file ivag059_supplementary_data.zip › Supplementary Material 1.pdf]

Activated Clotting Time

Dear Colleagues,

We invite you to participate in a questionnaire aimed at gathering information on the methodologies applied in the management of Activated Clotting Time (ACT) testing in cardiac surgery centers across Europe. The focus of this survey is specifically on the management of ACT following reversal with protamine.

This questionnaire is intended for use in adult cardiac surgery, non-emergent procedures, and on-pump surgeries (with cardiopulmonary bypass).

Your insights and experiences are invaluable and will contribute significantly to the understanding and improvement of ACT management practices in cardiac surgery. This survey follows the recent publication of my paper (link), and aims to build on the findings presented therein.

Thank you for your time and contribution to this important study.

Best regards,

Rafael Maniés Pereira
Cardiopneumologist - Perfusionist
Biostatistician, Data Analyst

* Indica uma pergunta obrigatória

1. Hospital name *

2. Country *

3. Number of **on-pump heart surgeries** performed annually in your hospital? *

Marcar apenas uma oval.

- < 100
- 100-300
- 301-500
- >500

4. Is the surgery performed with normothermic or hypothermic circulation? *

Marcar apenas uma oval.

- Normothermia (>35°C)
- Mild hypothermia (32-35°C)
- Moderate hypothermia (28-32°C)
- Deep hypothermia (<28°C)

Heparine Administration and target-ACT

5. Is there a written protocol in your hospital center where heparinization is described? *

Marcar apenas uma oval.

- Yes
- No

6. What is the device used for monitoring ACT? *

____

7. Do you use any device for quantifying the dose-response of heparin? *

Marcar apenas uma oval.

- Yes
- No

8. What dose of heparin is **routinely** administered before starting CPB? *

Marcar apenas uma oval.

- 200 UI/Kg
- 250 UI/Kg
- 300 UI/Kg
- 350 UI/Kg
- 400 UI/Kg

9. What is the target value of ACT after heparin administration and before on-pump entry? *

Marcar apenas uma oval.

- 400 sec.
- 450 sec.
- 480 sec.
- Outra: ______________________

10. How often do you measure ACT during CPB? *

Marcar apenas uma oval.

- Every 30 minutos
- Every 60 minutes
- Only when clinically indicated
- Outra: ______________________

11. What method do you use to monitor heparin levels during CPB? *

Marcar apenas uma oval.

- Activated Clotting Time (ACT)
- Heparin concentration (e.g., Hepcon HMS)
- Anti-Factor Xa levels
- Others

12. Does the team follow any specific strategy for managing heparin in high-risk bleeding patients (e.g., renal failure, redo surgery, minimally invasive cardiac surgery)?

Marcar apenas uma oval.

- Yes
- No

13. If you answered **yes** to the previous question, please specify?

____

14. What is the strategy for suspected heparin resistance (e.g., reduced antithrombin III levels)?

Marcar apenas uma oval.

- Administration of AT-III
- Increased heparin dose
- Use of bivalirudin or another alternative anticoagulant
- No specific protocol

Protamine Administration

15. Is the protamine dose adjusted according to any specific variable? *

Marcar apenas uma oval.

- Patient weight
- CPB duration
- Total heparin dose administered
- Fixed dose based on protocol

16. What is the protocol for calculating the dose of protamine administered after weaning from CPB?

Marcar apenas uma oval.

- <1:1
- 1:1
- >1:1

17. How is protamine administered? *

Marcar apenas uma oval.

- Bolus
- Slow infusion
- Slow infusion (diluted)
- Case-dependent

18. What is the frequency of complications related to protamine administration at your center (e.g., severe hypotension, anaphylactic reaction, pulmonary hypertension)?

Marcar apenas uma oval.

- Very low
- Low
- Moderate
- High

19. How long do you wait **after administering protamine** before collecting the blood sample for ACT measurement?

Marcar apenas uma oval.

- < 5 minutes
- 5 minutes
- > 5 minutes

20. Is the target ACT value protocolled after reversal with protamine? *

Marcar apenas uma oval.

- Yes
- No

21. Is there any universal numerical value of ACT to be achieved by all patients after reversal with protamine (Example: ACT < 140 seconds)?

Marcar apenas uma oval.

- Yes
- No

22. **If you answered yes** to the previous question, what is this value?

____

23. Are any of the following options used in your hospital to affirm that the ACT value is **complete or satisfactory** after reversal with protamine?

Marcar apenas uma oval.

- ACT value below baseline ACT
- ACT value close to baseline ACT
- None

24. Would you consider administering another dose of protamine at any ACT value? *

Marcar apenas uma oval.

- Yes
- No, an additional dose of protamine is never administered

25. If you answered yes to the previous question, what is this value?

____

26. If you administer an additional dose of protamine, what is the main reason for this decision?

Marcar apenas uma oval.

- There is an ACT value at which a booster dose is administered
- There is an ACT value at which a booster dose is administered, but only if the patient has difficulties with hemostasis.
- Only if the patient has difficulties with hemostasis, regardless of the ACT value
- None

27. When administering a protamine booster dose, what is the correct dosage to be administered?

____

28. Does your hospital have a thromboelastogram (TEG or ROTEM)? *

Marcar apenas uma oval.

- Yes
- No

29. **If yes**, is this device in the operating room or another service (e.g. Blood Service or Analys Laboratory)?

Marcar apenas uma oval.

- Operating room
- Another Service

30. How is the decision made to administer blood products? *

Marcar apenas uma oval.

- Based solely on ACT
- Based on TEG/ROTEM
- Based on clinical criteria from the surgeon and/or anesthesiologist
- Outra: ______________________

31. Has your hospital recently implemented any changes to ACT/protamine protocols based on new scientific data?

Marcar apenas uma oval.

- Yes
- No

32. If you answered **yes** to the previous question, please specify?

____

Este conteúdo não foi criado nem aprovado pela Google.

Google Formulários
